# A manual-based family intervention for families living with the consequences of traumatic injury to the brain or spinal cord: a study protocol of a randomized controlled trial

**DOI:** 10.1186/s13063-019-3794-5

**Published:** 2019-11-27

**Authors:** Pernille Langer Soendergaard, Mia Moth Wolffbrandt, Fin Biering-Sørensen, Malin Nordin, Trine Schow, Juan Carlos Arango-Lasprilla, Anne Norup

**Affiliations:** 1 Department of Neurorehabilitation, TBI Unit, Rigshospitalet, Kettegaard Allé 30, 2650 Hvidovre, Denmark; 2 Department of Psychology, University of Southern Denmark, Campusvej 55, 5230 Odense, Denmark; 3 Clinic for Spinal Cord Injuries, Rigshospitalet, University of Copenhagen, Havnevej 25, 3100 Hornbæk, Denmark; 4 Institute for Clinical Medicine, University of Copenhagen, Blegdamsvej 3B, 2200 Copenhagen, Denmark; 5 Department of Neurosurgery, Karolinska University Hospital, Eugeniavägen 3, 171 76 Stockholm, Sweden; 6 Research and Development, Brain Injury Center BOMI, Maglegaardsvej 15, 4000 Roskilde, Denmark; 7 BioCruces Vizcaya Health Research Institute, Cruces University Hospital, Barakaldo, Spain; 8 IKERBASQUE, Basque Foundation for Science, Bilbao, Spain; 9 Department of Cell Biology, University of the Basque Country (UPV/EHU), Leioa, Spain

**Keywords:** Traumatic brain injury, Traumatic spinal cord injury, Family intervention, Randomized controlled trial, Study protocol, CONSORT-SPI 2018

## Abstract

**Background:**

Acquiring a traumatic injury constitutes a severe life change for the survivor, but also for the surrounding family. The paradigm of helping the family has primarily been on psychosocial interventions targeting caregivers. However, interventions including both survivor and caregivers should be an essential part of treatment, as the whole family’s functional level and mental health can be affected. The current study protocol presents a manualized family intervention for families living with traumatic injury to the brain (TBI) or spinal cord (tSCI). The objectives are to investigate if the intervention improves quality of life (QoL) and decreases burden. It is hypothesized that the family intervention improves problem-solving strategies and family dynamics, which will reduce the burden. This may improve the caregivers’ mental health, which will improve the support to the survivor and QoL.

**Methods:**

The study is an interventional, two-arm, randomized controlled trial. During a 2-year period, a total of 132 families will be included. Participants will be recruited from East-Denmark. Inclusion criteria are (1) TBI or tSCI, (2) ≥ 18 years of age, (3) ≥ 6 months to ≤ 2 years since discharge from hospital, (4) ability to understand and read Danish, (5) cognitive abilities that enable participation, and (6) a minimum of one family member actively involved in the survivor’s life. Exclusion criteria are (1) active substance abuse, (2) aphasia, (3) prior neurologic or psychiatric diagnose, and (4) history of violence. Within each disease group, families will be allocated randomly to participate in an intervention or a control group with a ratio 1:1. The intervention groups receive the family intervention consisting of eight sessions of 90 min duration. Families in the control groups receive 2 h of psychoeducation. All participants complete questionnaires on QoL, self-perceived burden, family dynamics, problem-solving strategies, mental health, and resilience at pre-intervention, post-intervention, and 6-month follow up.

**Conclusion:**

If the intervention is found to have effect, the study will contribute with novel knowledge on the use of a manual-based intervention including the entire family. This would be of clinical interest and would help families living with the consequences of TBI or tSCI.

**Trials registration:**

ClinicalTrials.gov, NCT03814876. Retrospectively registered on 24 January 2019.

## Background

### Background and objectives

Each year traumatic injury to the brain (TBI) or spinal cord (tSCI) affects approximately 3000 individuals and their families in Denmark [1, 2]. TBI and tSCI are complex injuries often followed by a broad range of disabilities. Survivors of TBI may experience cognitive, somatic, affective, behavioural, and motor difficulties [3, 4], and survivors of tSCI may experience complete paralysis or motor and sensory difficulties in relation to urinary bladder, bowel management, sexual function, respiratory and cardiovascular function etc. [5]. Acquiring a traumatic injury not only has an impact on the survivor, but will also affect their families, as they will have to adapt to the changed life situation as well [6–8]. The consequences can affect the family early on [9, 10], but also long term [11–13]. Several families report low level of family functioning up to 5 years after injury [14, 15]. Very often the family members must perform a considerable number of tasks related to the patient. About 43% of caregivers for TBI survivors report spending more than 5 h per week helping their injured family member, and of this group, 22% spend more than 16 h each week [11, 12]. In a group of caregivers for patients with tSCI, 39% reported they stopped working to be able to take care of their injured family member [16]. Families with TBI report high frequencies of anxiety and depression symptoms [17, 18], and high emotional burden [7, 13]. Similar results have been found among families living with tSCI [6, 19]. Furthermore, impaired quality of life (QoL) has been reported for both groups [5, 10, 12, 13, 16, 20], for as long as up to 20 years after injury [21]. The reason may be that the family is facing a new life situation for which they are not prepared, as a traumatic injury is sudden and unexpected [22]. Consequently, most families do not have the appropriate coping strategies [23–25]. Thus, it is important to help the family in coping with the changed life situation [26].

So far, the paradigm and focus of helping the family has been psychosocial interventions primarily targeted at caregivers. The assumption behind most interventions has been that the best way to take care of the survivor is to take care of the caregiver. Thus, most interventions have included only the primary caregiver of the close family. Such interventions have consisted of psychoeducation and support [27–30], stress management [18, 31] skill-building and problem-solving strategies [32–35]. These interventions have shown improvement of problem-solving strategies, reduced emotional burden, anxiety and depression for the primary caregiver. However, it is unclear how this type of intervention affects the survivor. As most intervention programmes work with the survivor and caregiver separately, there is a lack of focus on the entire family. Interventions including the whole family should be an essential part of the treatment [36], as the injury can affect the family dynamics, including roles, boundaries, and communication. Both internationally [19, 24, 37–39] and nationally in Denmark [7, 9, 11, 40], negative effects on the family’s functional level and mental and physical health have been well-documented. This can affect the quality of the care that the caregiver provides [8]. By intervening in the functioning of the entire family, it may be possible to influence QoL and the outcome for both the survivor and the caregiver [8]. However, such a relationship has not yet been elucidated longitudinally, and there have been only a few cross-sectional studies [25, 41].

In a pilot study conducted in Latin America [42], the effectiveness of a newly developed eight-session manualized family intervention for individuals with spinal cord injury (SCI) was evaluated. The preliminary results of this pilot study showed large effect sizes in relation to depression, anxiety, stress and problem-solving strategies. Families, who received the intervention, experienced significant improvements in depression and anxiety, and this effect was maintained at 6-month follow up. Furthermore, effects on burden were also found as well as improved problem-solving strategies. Based on these promising pilot results, this current study seeks to investigate the family intervention in a Danish population.

Consequently, the aim of the present study is to investigate the effect of the eight-session manualized family intervention on QoL in individuals with TBI or tSCI and their family members, compared to a control group. The effect will be measured short term (post-intervention) and long term (6-month follow up). It is assumed that people who survive a traumatic injury do so in the context of their family. Therefore, a minimum of one family member, actively involved in the patient’s life, must be included in each session of the intervention. To our knowledge, the present randomized controlled trial (RCT) will be the first to evaluate the effectiveness of a manual-based family intervention to improve QoL in the entire family. This will be investigated by the following primary research question: Will the families in the intervention groups report improved QoL compared to the families in the control groups? The secondary research question is: Will the families in the intervention groups report decreased burden compared to the families in the control groups? It is hypothesized that the family intervention will lead to improved QoL and decreased burden for both the survivor of TBI or tSCI and their caregivers. Consequently, better mental health of the caregiver will improve the care and support they provide to the survivor. This improvement will ultimately improve the QoL of the survivor.

## Methods

### Trial design

**Fig. 1:**
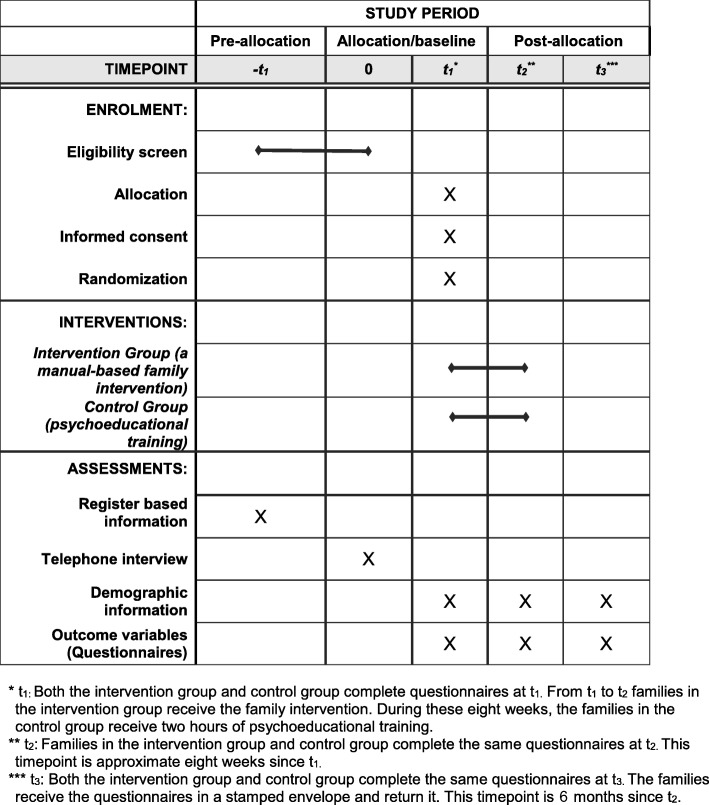
Standard Protocol Items: Recommendations for Interventional Trials (SPIRIT) table of enrolment, intervention, and assessments

This is a two-arm RCT of a manualized family intervention conducted in two different disease groups: (1) patients with TBI and (2) patients with tSCI, participating with a minimum of one family member actively involved in the patient’s life. The duration of the study is from October 2018 to October 2021 with an inclusion period of 2 years. The trial was developed according to the guidelines for Standard Protocol Items: Recommendations for Interventional Trials (SPIRIT) statement [43] (see Additional file 1 and Fig. 1), and will be reported as stated in the Consolidated Standards of Reporting Trials statement for reporting randomized trials of social and psychological interventions (CONSORT-SPI) [44].

### Participants

The present study has an uptake area covering the eastern part of Denmark including the Region Zealand and Capital Region with a total of almost 46% of all inhabitants in Denmark [45]. Patients from this part of Denmark, who have either TBI (moderate to severe) or tSCI, can be included with a minimum of one family member if meeting the following criteria:

1. Age ≥ 18 years at time of inclusion in the study
2. Between ≥ 6 months and ≤ 2 years since discharge from hospital
3. Ability to understand and read Danish
4. Patients with TBI must score ≥ 7 on the Rancho Los Amigos Scale at time of inclusion in the study, indicating the resolution of post-traumatic amnesia (PTA)
5. Cognitive abilities that enable participation in a manualized intervention (when in doubt, the patient must have Mini Mental State Examination score ≥ 23)

Family members must meet the following criteria:

1. Actively involved in the patient’s life
2. Age ≥ 18 years at time of inclusion in the study
3. Ability to understand and read Danish

Patients and family members will be excluded if they meet any of the following criteria:

1. Active substance abuse
2. Severe aphasia
3. Prior diagnosis of neurologic or psychiatric disorder
4. History of violence in the family

#### Recruitment and informed consent

The research study will be conducted at the Department of Neurorehabilitation, TBI Unit, Rigshospitalet, Denmark. The participants will be recruited from two different clinics in Denmark: one clinic for patients with TBI and one clinic for patients with tSCI. Two project nurses will recruit participants, provide information about the study, randomize, and obtain written informed consent. All family members are informed that they have the right to withdraw their consent at any time during the study period. In the case of withdrawal, the family is asked if data already collected can be included in future analysis.

### Manualized family intervention

In the present study, the manualized intervention “Traumatic Brain Injury (TBI)/Spinal Cord Injury (SCI) Family Intervention” will be used [46]. The whole manual for conducting the intervention has been published by the developers and can be accessed from the online version (doi:10.1310/sci2201-49) of their pilot study in the supplementary material [42].

The intervention was developed for patients with TBI or tSCI and their families with the overall purpose to improve the functioning of the individual and the family. The intervention was based on clinical experience and empirical research and constructed by a neuropsychologist in collaboration with a family therapist and a psychologist specializing in cognitive behavioural therapy [46]. In 2017, the manual was translated into Danish by authors TS and AN, and all questions concerning the intervention and translation process were discussed with the developers.

The intervention consists of a 90-min session once a week for 8 weeks and includes strategies and elements from both couples and family therapy. Session 1 is facilitated by the nurse and all participants (both intervention and control groups) will be supervised, while completing the questionnaires before randomization. Sessions 2–8 will address different topics or special strategies and are facilitated by a trained neuropsychologist. Between each session the participants will complete a homework assignment, a between-session task, where the new strategies can be practiced. The content of the sessions is structured as follows (Table 1) [42].

**Table 1:** An overview of the sessions, the content and the frequency for the intervention group

Week	Session (topic)	Topic of discussion	Facilitator
1	Introduction	Information about the study; consent to participation; completion of baseline questionnaires; randomization; The homework assignment is given (90 min)	Nurse. If the family is randomized to the intervention group at the end of the session, the neuropsychologist will present the homework assignment
2	Making meaning	Presentation of the study and the individual sessions; expectations to the participants and the facilitator; making meaning of TBI/tSCI and psychoeducation; the homework assignment is reviewed and a new given; session rating scale (90 min)	Neuropsychologist
3	Shifting focus	The relationship between thoughts, feelings, and behaviour; the homework assignment is reviewed and a new given; session rating scale (90 min)	
4	Managing emotions	Learning to identify signs indicating an escalation of emotions and techniques to handle emotions; strategies for overcoming negative emotions; the homework assignment is reviewed and a new given; session rating scale (90 min)	
5	Communicating effectively	Talk-listening techniques and communication improving strategies; communication danger sings; the homework assignment is reviewed and a new given; session rating scale (90 min)	
6	Finding solutions	From problem-talk to solution-talk; problem-solving strategies; the homework assignment is reviewed and a new given; session rating scale (90 min)	
7	Boundary making	Understanding the importance of boundaries in the family; self-care; healthy family dynamics; the homework assignment is reviewed and a new given; session rating scale (90 min)	
8	Conclusion and farewell	Summary of learned skills; the homework assignment is reviewed; session rating scale; completion of post-intervention questionnaires (the facilitator leaves the room) (90 min)	
34 (6 months after session 8)	Follow up	Completion of the follow-up questionnaires. The families receive the questionnaires in a stamped envelope. The families return the questionnaires to the nurse. They will receive a reminder if they forget to return the questionnaires and a follow up by phone if no answer	Nurse

Each session of the intervention follows the same structure (except for session 1: Introduction). The session begins with a quote relevant for the topic of the session. Afterwards, the homework assignment will be evaluated. A new topic is then presented. New techniques and strategies will be practiced and used in relation to a specific challenge faced by the family. Finally, a new homework assignment will be presented.

The family intervention aims at helping families [46] to (1) share ideas about and experiences of the traumatic injury and to create a greater understanding of each other; (2) elicit misunderstandings about a traumatic injury; (3) change mindsets from negative to more positive aspects of their situation; (4) recognise the influence their thoughts have on their emotions; (5) identify signs that indicate escalation of emotions and to learn strategies to manage their emotions; (6) communicate more effectively; and (7) understand the importance of boundaries in the family and learn to clarify them together [46].

### Control group

Families allocated to the control group will participate in one psychoeducational session of 2 h, either individually or in a group. This will take place within the first 8 weeks after baseline. The facilitator will be an experienced neuropsychologist, who is not involved with the intervention groups. The content of the 2-h session will be equal for all participants in the control groups. They will receive information about the consequences of a traumatic injury, and how it can affect the entire family. Furthermore, information on normal emotional reactions, both in the acute phase, but also in long term, will be discussed. At the end of the session, the family members will be able to share their experiences, and the facilitator will give them advice about, where to receive more support and/or information. The control groups are offered psychoeducational support as it seems unethical not to offer these families anything because of the promising results from the pilot study in Latin America [42]. The rationale behind offering these families a single psychoeducational session is that they will receive some support, but the support is expected to have a short-term effect and will consequently not affect the results when comparing the two groups (intervention group and control group). If the family intervention turns out to be effective, all families allocated to the control groups will be offered the 8-week family intervention after the study completion.

### Outcomes

The manual-based family intervention for families with TBI or tSCI has not been implemented in Denmark prior to this study. Thus, we are interested in the effect of the family intervention, but also the families’ subjective experiences of participating in the intervention. Consequently both quantitative and qualitative data will be collected.

#### Questionnaires

##### Socio-demographic data

The following information will be collected at baseline (session 1): age, gender, number of years of education, and cause and date of injury. Other socio-demographic characteristics will be collected at all three timepoints, i.e. baseline, post-intervention (8 weeks after baseline) and at 6-month follow up including civil status, family member relationship, duration of relationship, members in the household, employment status, number of weekly hours spent on work or education, comorbidities, medical drug use, social network, and whether they have participated or currently are participating in another therapeutic setting with a psychologist. The patients will also complete questions about rehabilitation, if they still receive support from an occupational or physio therapist, and if they have been hospitalized during the family intervention. Caregivers will complete information on the hours spent on caring/supervising the patient each day.

##### Injury characteristics

The Glasgow Coma Scale [47] and length of PTA [48, 49] will be used as an indicator of injury severity for patients with TBI. The neurological level and severity of the spinal cord lesion will be assessed in patients with tSCI according to the International Standards for Neurological Classification of Spinal Cord Injury, including the American Spinal Injury Association Impairment Scale [50, 51]. The length of stay will be registered for both disease groups.

##### Primary and secondary outcome

The effect of the family intervention will be measured by self-report questionnaires. The outcome measurements are outlined in Table 2 and will be measured at baseline, post-intervention, and at 6-month follow up (Table 2).

**Table 2:** Primary and secondary outcome measures

Domain	Assessment	Test tool (outcome measure)	Administered by
Quality of life (QoL)	Generic QoL	Short Form-36 (SF-36) [9, 10, 52, 53]	All participants
	The patient’s disease-specific QoL	QoL after Brain Injury (Qolibri) [54]	Participants with TBI
		International Spinal Cord Injury QoL Basic Data Set [55]	Participants with tSCI
Self-perceived burden	General burden, isolation, disappointment, emotional involvement, environment	Caregiver Burden Scale (CBS) [12, 56]	Caregivers
	Physical, emotional and financial burden	Self-Perceived Burden Scale (SPB) [57]	Participants with TBI or tSCI
Family dynamic	Flexibility	Family Adaptability and Cohesion Evaluation Scales-IV (FACES-IV) [58]	All participants
	Cohesion		
	Communication		
Problem-solving abilities	Confidence	Problem-Solving Inventory (PSI) [59]	All participants
	Personal control		
	Approach-avoidance		
Mental health	Depression	Patient Health Questionnaire-9 (PHQ-9) [60]	All participants
	Anxiety	Generalized Anxiety Disorder-7 (GAD-7) [61]	
	Satisfaction with life	Satisfaction with Life Scale (SWLS) [62]	
Relationship	Relationship to relatives	Relationship Assessment Scale (RAS) [63–65]	All participants if a dyad
Resilience	Resilience and robustness	The Resilience Scale for Adults (RSA) [66]	All participants

All participants in the two groups will complete self-report questionnaires to collect generic QoL data as the primary outcome measurement, and self-perceived burden, family dynamics, problem-solving strategies, mental health, and resilience as secondary outcome measurements. In cases where the family consists of a dyad, e.g. a married couple, mother and daughter etc., rather than several family members, the Relationship Assessment Scale (RAS) [63–65] will be completed as a supplement.

##### Treatment compliance

All participants and the neuropsychologist will complete a measure of compliance. Participants allocated to the intervention group will individually complete the Session Rating Scale (SRS) [67] after each session. The SRS is a paper and pencil four-item scale used to evaluate the individuals’ experience with the relevance of the specific topic of the session, including strengths and weaknesses, and their experience with the neuropsychologist. The neuropsychologist will leave the room during this task. The families will be instructed to put the completed questionnaire in an envelope, consequently the neuropsychologist will be blinded to their answers.

When the 8 sessions are completed, the neuropsychologist will complete an evaluation form, the Therapist Checklist Scale, which evaluates the participants’ attendance [68]. It is a five-item scale, where the neuropsychologist will rate the participants according to their engagement in the intervention, including level of participation, homework assignments, interaction with the other family members and the neuropsychologist, and their ability to use the strategies from each session [68, 69].

#### Semi-structured interviews

The experiences with the manual-based family intervention will be investigated qualitatively by conducting individual semi-structured interviews with 20 families who have finished the 8-week family intervention. The families will be selected based on representativeness of disease (TBI or tSCI), different family structures (e.g. couples, parents with adult children, siblings), and age and gender of the survivor. Qualitative data will be used to analyse, it will be possible to add qualitative data, which will be used to analyse the families’ individual experience of participating in the intervention. The interviews will include the families’ experiences of the structure and content of each session, and the amount and content of homework. Furthermore, the participants will be asked if and how the intervention was meaningful for their specific situation. A research nurse will conduct the interviews within the first 4 weeks after the intervention has been completed will interview the families within the first 4 weeks after the intervention has been completed.

### Sample size

Preliminary statistical power calculations have been carried out to allow us to detect any group effect (intervention versus control in the TBI and tSCI group, respectively) on the primary outcome variable, i.e. generic QoL data assessed by the Short Form-36 (SF-36) [9, 10]. Numbers of participants required in the study were calculated based on the Mental Component Summary (MCS) of the SF-36 [52, 53]. As no studies have been carried out in Denmark using the SF-36 in patients with a traumatic injury, a Norwegian study [70] was used for the sample size calculations. In the Norwegian study, a score of 43.8 (SD 12.5) was reported among patients with moderate to severe TBI. Based on a confidence level of 95%, a power level of 80%, and a significant difference of 5 points between the intervention and control group, it will be necessary to recruit 182 participants to each arm of the trial i.e. the TBI and tSCI arms. To account for a 10% drop-out rate, 400 participants in total must be included.

### Randomization

#### Sequence generation and allocation concealment mechanism

Within each disease group, families will be allocated randomly to the intervention group or the control group with an allocation ratio of 1:1. The Sealed Envelope application will be used to generate random allocation sequences: this is an online software application for randomizing patients into clinical trials [71]. The random allocation sequence will be generated and concealed from the employees in the project. The families will consecutively be allocated to each group. Two arms for the TBI group and two arms for the tSCI group will be created with 66 families in each disease-specific group. The randomization will be conducted in blocks of 22, which increases the likelihood that the intervention groups and the control groups will have the exact same number of participants, even in the case of recruiting fewer participants than expected. When entering the unique family ID number in Sealed Envelope, the application randomizes the family to either the intervention group or the control group.

#### Implementation and awareness of assignment

The project nurses will be responsible for identifying and recruiting families that meet the inclusion criteria. The random allocation sequence will be generated by the nurses using the online randomization procedure [71]. Families will complete the baseline questionnaires before randomization.

Because of the nature of the study, it will be clear to all participants and the neuropsychologist whether the families are allocated to the intervention group or the control group. It is not possible to blind the participants or the neuropsychologist to the group allocation. The nurses will collect the study data. The neuropsychologist and statistician performing the data analyses will not be involved in collection of data on the outcome measures. Thus, they will be blinded to the association between the data from the questionnaires and the group to which the families are allocated. The nurses who are responsible for the data collection will not participate in data analysis.

#### Analytical methods

Data will be stored in a password-protected electronic database, REDCap [72], which is a web-application for building and managing databases. All participants will be assigned a unique trial ID number and all demographic data, and data from the files and questionnaires will be stored in the database to ensure quality of the data. The nurses will be responsible for this part of the data management process.

The following analyses will be conducted in each arm of the trial. First, the baseline level of the primary outcome, generic QoL data, will be compared between groups using one-way analysis of variance (ANOVA). If there are significant differences at baseline, we will adjust for these in the following analyses. Intention-to treat (ITT) analyses will be performed to examine the effect of the family intervention using repeated measures ANOVA (rANOVA), with “time” as the repeated measurement. The comparison between the intervention group and control group in each disease group will be the between-group factor. Baseline characteristics will be assessed and compared between groups using one-way ANOVA, and we will adjust for these characteristics if necessary.

Little’s Missing Completely at Random (MCAR) approach will be applied for analysis of group allocation and of disease group to investigate whether drop-outs or missing data are associated with one of these parameters. Data analysis will be completed using STATA.

## Discussion

The objective is to investigate the effectiveness of the Danish version of the manualized family intervention for families living with TBI or tSCI. The project is innovative as it includes all family members in the intervention. The randomized controlled design will provide knowledge about the effectiveness of the intervention, and furthermore the semi-structured interviews will give insight into the individual experiences of the participating families. This study will provide contributions to the literature on the importance of including the whole family in the intervention. The manual-based family intervention addresses the family function, including both the survivor of TBI or tSCI and the closest family members.

If the intervention is evaluated as having an effect, the aim is to make the family intervention a permanent programme. If we identify an effect of the manual-based family intervention on the primary outcome measure, the aim is to strengthen efforts towards helping patients in these disease groups and their families in Denmark. This can involve cross-sectoral partnerships between the hospital departments and specific municipalities in Denmark, and can include training of professionals working in rehabilitation. With this project, the aim is to ensure that survivors of a traumatic injury and their families will be helped to regain the structure and function in the family, and consequently experience improved health.

### Limitations and generalizability

The present study will have some limitations that should be addressed. First, the study will only include families, who are willing to participate. Consequently, some families, who would fulfill our inclusion criteria, will not be included. One could speculate which families will be willing to participate in the study. Will it be families, who need help and support, or families, who have the time and resources? If a family refuses to participate, the reason for refusal will be obtained. Furthermore, as participation will be voluntary, all family members have the right to withdraw their consent if they want to quit the project. One could speculate whether families allocated to the control group will be more likely to drop out compared to families in the intervention group, because of a great desire to receive help. However, as the family intervention is very intense and time-consuming, this can also affect the risk of drop-outs in the intervention group. Therefore, prior to the allocation the nurses will inform the families about the content of the intervention in both groups both orally and in writing.

Second, most patients included in the study will have been hospitalized for specialized neurorehabilitation due to a severe traumatic injury. However, patients with severe cognitive disabilities or severe aphasia will be excluded, as patients with such disabilities will not be able to participate fully in the family intervention. This will affect the generalizability of the study, as patients with the most severe injuries or sequela will be excluded.

Third, participants will be recruited from only two clinics in Denmark - one clinic for patients with TBI and one clinic for patients with tSCI. This warrants caution with respect to generalizing the results and could potentially limit the external validity. However, as the two clinics have an uptake area covering the whole eastern part of Denmark with a total of almost 2.6 million inhabitants [45], the representativeness and generalizability is expanded.

Finally, due to the study design, it will not be possible to blind the participants or the neuropsychologist to the allocation. However, the researchers responsible for statistically analysing baseline, post-intervention, and follow-up questionnaire data will not know to which group the families were allocated.

### Interpretation

For families allocated to the intervention group, there can be some benefits and harms to consider. Participating in the family intervention may be costly for some families in terms of resources and time rather than an opportunity to receive support and help. This may influence the willingness to participate in the 8-week intervention, as participation may seem too time-consuming. To minimize the burden for the families, the neuropsychologist and nurses will be flexible in scheduling the sessions and the location of the intervention, e.g. if the family members have work or study obligations, then it will be possible to schedule a late session, and if the patient suffers from severe fatigue, then the intervention can be offered at home. Furthermore, the families can have transport expenses reimbursed and the intervention is free of charge.

For families allocated to the control group, there can be some ethical considerations. All families are carefully informed about the study design prior to inclusion, including the information that the randomization to either the intervention group or the control group is 50:50. However, it is possible that families who are willing to participate in the project hope to be allocated to the intervention group. In the worst case, families allocated to the control group may feel rejected, as they had high hopes of receiving help and support. This may harm the family and make them withdraw their consent and quit the project. However, the families allocated to the control group will be offered one psychoeducational session with a neuropsychologist.

## Important information

### Registration

ClinicalTrials.gov Identifier: NCT03814876, registered on 24 January 2019.

### Protocol

The Protocol Record 2018_0004, Family Intervention Following Traumatic Injury is accessible to the public on ClinicalTrials.gov.

### Declaration

The project is funded by The Council of Danish Victims Fund (grant 18–610-00024) and Foreningen Oestifterne (j.nr. 18–077). The funders have no influence on the design of the study, on data collection or analysis, and did not participate in the writing of the manuscript. The authors declare no other potential interests.

### Stakeholder involvement

Stakeholders in the project are amongst others the clinics from where participants will be recruited. Furthermore, the patient organizations in Denmark may have an interest in the results of the project. However, the clinics and the patient organizations will not be involved in the trial design, conduct or analyses, and there will be no incentives offered.

Juan Carlos Arango-Lasprilla (JCAL) is one of the developers of the manual-based family intervention for families living with TBI or tSCI. He is a part of this research group, currently investigating the effect of the Danish version of the manualized family intervention.

## Trial status

This is study protocol version 1.0. Recruitment of participants for the study commenced in September 2018, and the first family was included and participated in session 1 on 17 October 2018. At present, 13 families have been randomly assigned (8 families of a patient with TBI and 5 families of a patient with tSCI): 5 families have been randomized to the intervention group and 8 families have been randomized to the control group; currently, 4 families have participated in the semi-structured interview. The estimated enrollment period, including recruitment and inclusion, is anticipated to be 2 years (October 2020) and will conclude, when the estimated sample size has been included.

## Supplementary information

**Additional file 1.** SPIRIT 2013 Checklist: Recommended items to address in a clinical trial protocol and related documents.

## Data Availability

The data materials are available from the corresponding author on reasonable request if the Danish Data Protection Agency accepts such request.
