# Correction to: A manual-based family intervention for families living with the consequences of traumatic injury to the brain or spinal cord: a study protocol of a randomized controlled trial

**DOI:** 10.1186/s13063-019-3978-z

**Published:** 2019-12-27

**Authors:** Pernille Langer Soendergaard, Mia Moth Wolffbrandt, Fin Biering-Sørensen, Malin Nordin, Trine Schow, Juan Carlos Arango-Lasprilla, Anne Norup

**Affiliations:** 1 Department of Neurorehabilitation, TBI Unit, Rigshospitalet, Kettegaard Allé 30, 2650 Hvidovre, Denmark; 2 Department of Psychology, University of Southern Denmark, Campusvej 55, 5230 Odense, Denmark; 3 Clinic for Spinal Cord Injuries, Rigshospitalet, University of Copenhagen, Havnevej 25, 3100 Hornbæk, Denmark; 4 Institute for Clinical Medicine, University of Copenhagen, Blegdamsvej 3B, 2200 Copenhagen, Denmark; 5 Department of Neurosurgery, Karolinska University Hospital, Eugeniavägen 3, 171 76 Stockholm, Sweden; 6 Research and Development, Brain Injury Center BOMI, Maglegaardsvej 15, 4000 Roskilde, Denmark; 7 BioCruces Vizcaya Health Research Institute, Cruces University Hospital, Barakaldo, Spain; 8 IKERBASQUE Basque Foundation for Science, Bilbao, Spain; 9 Department of Cell Biology, University of the Basque Country (UPV/EHU), Leioa, Spain

**Correction to: Trials (2019) 20:646**

**https://doi.org/10.1186/s13063-019-3794-5**

After publication of our article [1] we were notified that a few duplicate sentences were included on page 7 of the manuscript.

The original article has been corrected.
